# The Need for Centralization of Computational Biology Resources

**DOI:** 10.1371/journal.pcbi.1000372

**Published:** 2009-06-26

**Authors:** Fran Lewitter, Michael Rebhan, Brent Richter, David Sexton

**Affiliations:** 1 Bioinformatics and Research Computing, Whitehead Institute for Biomedical Research, Cambridge, Massachusetts, United States of America; 2 Novartis Institutes for BioMedical Research, Basel, Switzerland; 3 Enterprise Research IS and Informatics, Brigham and Women's Hospital, Massachusetts General Hospital, and Partners Healthcare, Boston, Massachusetts, United States of America; 4 Center for Human Genetics Research, Computational Genomics Core, Vanderbilt University, Nashville, Tennessee, United States of America

Biomedical research is benefiting from the wealth of new data generated in the laboratory through new instrumentation, greater computational resources, and massive repositories of public domain data. Using these data to make scientific discoveries is sometimes straightforward, but can be complicated by the number and breadth of public sources available to the researcher as well as by the plethora of tools from which to choose. Complex searches, analyses, or even storage needs require more computational expertise than that available within an individual laboratory. As biomedical researchers develop more computational skills, this may change over time. Having a centralized group of experts in computational biology can be of great value to the experimental biologist, and, recognizing this, many organizations have invested in building a team of computational biologists, bioinformaticists, and research IT services to address the needs of the investigators. This Editorial presents our views on the benefits and challenges of centralizing these activities.

In order to benefit from expertise among existing teams of experts around the world, the “Bioinfo-Core” group was formed during the ISMB 2002 meeting in Edmonton, Canada, with approximately 25 initial members. Since then, the group has expanded in both organization and interest. Our worldwide membership now includes more than 150 people who administer centralized bioinformatics and research computing facilities within diverse organizations, including academia, independent research institutes, academic medical centers, and industry. Additionally, the group holds quarterly meetings via teleconference, continues an annual face-to-face meeting at ISMB (averaging 40–60 people), and hosts a mailing list and Wiki (http://www.bioinfo-core.org) to further communication.

## Why Centralize?

Different institutions will have different names for these centralized resources—“core facility”, “platform”, etc.—and different responsibilities for the group based on size and organization. For the purposes of this Editorial and the accompanying Perspectives (doi:10.1371/journal.pcbi.1000368 and doi:10.1371/journal.pcbi.1000369), we use the term “Bioinformatics Core Facility” to refer to these centralized resources. No matter what name is used, the primary focus of the centralized resource will be to support the investigators with their computational needs. Below, we highlight some of the most important reasons we see for centralizing these resources.

### Providing Infrastructure

It is important for an institution to have a solid infrastructure for both hardware and software. This is especially true with respect to funding opportunities. Specifically, having a solid computational and bioinformatics infrastructure may increase the probability of a grant award whose main scientific exploration is heavily data-driven. Furthermore, funding agencies are offering larger, more integrated, complex, and cross-institutional projects. These grants do not fund de novo technical infrastructure, but most times provide incremental improvements to existing infrastructure. In addition, granting agencies find that centralizing resources is far more cost-efficient for large-scale projects. This is especially true for NIH Program Projects and Center grants, Clinical and Translational Science Awards, and for institutional or departmental research initiatives.

On the software side, it can be economical to purchase multi-user, concurrent, or site licenses rather than individual licenses. This also helps with support of the software as purchasers of the larger licenses will likely be better prepared to field questions and offer training opportunities about installation and use of the software. In addition, the Bioinformatics Core Facility may be in a position to purchase expensive software that is used only occasionally by researchers, thus being able to provide more options for individuals to address important research needs.

Many researchers in an institution may have the same needs for custom software. A person working in a centralized facility can identify such shared needs and build a robust tool for use by many researchers within the institution. These specialized tools or software functions can be reused, and this increases their value to the organization. It also prevents the multiple re-invention of solutions within institutions.

Furthermore, solutions developed and implemented within a centralized facility can be leveraged by institutional enterprise projects. Development, evaluation, and live testing of infrastructure or applications for a specific project need not be ad hoc in some cases. Frameworks can be developed that can translate to enterprise-wide applications providing competitive advantages in translational science activities. If effective, these technologies can be translated into the larger enterprise as-is, or, with adjustment, to fit within existing implementations, additional requirements, or vendor solutions.

### Staffing Issues

An important aspect of building a Bioinformatics Core Facility is hiring of staff. It is advantageous to do this as a centralized effort because it is easier for bioinformatics staff to understand and recognize the skills necessary for recruiting personnel. It is also helpful to have both senior and junior people in a group so that work can be distributed efficiently. A larger, centralized group can also offer mentoring and peer relationships.

Members of Core Facilities can develop skills and expertise in particular areas of bioinformatics to an extent that is difficult to achieve in environments where individuals are embedded in an individual investigator's research group—the core competencies of a larger team versus the narrow ability of a few individuals with multiple demands. Team efforts that combine the expertise of such core staff with different focus areas are often needed to address complex challenges at the forefront of science. Even if a lab has their own full-time bioinformatics scientists, they too can benefit from a central group in areas that are beyond their narrow focus or when their demand outpaces the lab's needs. Furthermore, for a given laboratory project, there are periods of intense work for bioinformatics staff and infrastructure interspersed with periods of calm. New researchers or early-stage projects will also benefit greatly from a centralized group. Having staff readily available to do preliminary analyses can help with funding opportunities. Therefore, building silos is inefficient and costly as the use of resources is not needed 100% of the time.

## Some Disadvantages of Centralization

Although we believe that the benefits far outweigh the risks, there are some issues to mention that may be seen as disadvantages of centralization. The major disadvantage to an individual lab may be loss of control over dedicated access to such resources as hardware, software, and personnel—resources typically provided by a centralized group. For example, if relying on a centralized facility for computational work, a lab may not have complete control over the person who is doing the work and may not have a dedicated person for their work. The person in the shared facility is likely to have other demands and needs around which to balance their priorities. Projects may not get completed as quickly as needed. Furthermore, since the person is not a lab member, they may be seen as “out of touch” with the scientific focus of the lab.

## Some of Our Challenges

In discussions among members of the Bioinfo-Core group, a number of challenges related to supporting the computational needs of scientists at institutions have emerged as common themes. Some of these challenges include the following.

1. How do we establish infrastructure for both IT and software? Depending on the structure of the institution, the relationship between IT and research computing will vary. Some organizations will put these focuses under one umbrella, while others will have them as separate. Having the group report on the science side (rather than administrative side) of an institution seems to work well. Either way, the two groups must coordinate to build a robust hardware and software environment to support the scientists.
2. How do we keep current as science and technologies move forward? The challenge here is to develop computational expertise in emerging science and new instrumentation. In addition, there is an ongoing need to evaluate new software and hardware tools and technologies for the experts and the end user.
3. How do we best train and educate scientists in bioinformatics concepts and best practices? Does this require formal courses? If so, what length? How frequent? What projects are better left to the experts, and what should experimentalists be doing?
4. How do we build a sustainable business and staffing model within the institution? Funding of a Bioinformatics Core Facility will vary from institution to institution, with some being fully funded by the institution itself and others relying on grants or chargeback models.
5. How do you build your “dream team” and provide an environment for growth and development of your staff? People who join Core teams often enjoy the challenge of working on many diverse projects rather than devoting their work to a specific project.
6. How is the Bioinformatics Core Facility evaluated? It might be based on how well its staff is integrated into laboratory research projects, how often staff are acknowledged in publications, and how many co-authored articles appear in high-profile journals.
7. How can the Bioinformatics Core Facility affiliate relevant non-Core members into the group? What role would these people have in the Core? This can broaden the scope of the Core.
8. How can the Bioinformatics Core Facility become involved in outreach? Through this mechanism, Cores can have an impact in addition to their primary responsibility of supporting the scientists in their institution.

These and others topics are addressed in the two accompanying Perspectives articles. The first Perspective discusses “Best Practices” for running a Bioinformatics Core Facility, primarily addressing ideas about building a well-integrated team (doi:10.1371/journal.pcbi.1000368). The second Perspective addresses how to respond to the changing scientific environment, particularly gearing up to support next-generation sequencing (doi:10.1371/journal.pcbi.1000369).

The content of these Perspectives has benefited greatly from the many discussions among the members of the Bioinfo-Core organization. We welcome new members and encourage those of you who are considering building a Bioinformatics Core Facility or are already running one to participate in our lively and useful discussions.

